# Tension force-induced bone formation in orthodontic tooth movement via modulation of the GSK-3β/β-catenin signaling pathway

**DOI:** 10.1007/s10735-017-9748-x

**Published:** 2017-12-09

**Authors:** Yelin Mao, Liangliang Wang, Ye Zhu, Yu Liu, Hongwei Dai, Jianping Zhou, Dechun Geng, Lin Wang, Yong Ji

**Affiliations:** 1 Nanjing Medical University, Nanjing, 211166 People’s Republic of China; 2 Suzhou Vocational Health College, Suzhou, 215009 People’s Republic of China; 3 The First Affiliated Hospital of Soochow University, Suzhou, 215006 People’s Republic of China; 4 Chongqing Medical University, Chongqing, 400016 People’s Republic of China

**Keywords:** Orthodontic tooth movement, Orthodontic force, Bone formation, Osteogenic differentiation, GSK-3β/β-catenin signaling pathway

## Abstract

Orthodontic force-induced osteogenic differentiation and bone formation at tension sites play a critical role in orthodontic tooth movement. However, the molecular mechanism underlying this phenomenon is poorly understood. In the current study, we investigated the involvement of the GSK-3β/β-catenin signaling pathway, which is critical for bone formation during tooth movement. We established a rat tooth movement model to test the hypothesis that orthodontic force may stimulate bone formation at the tension site of the moved tooth and promote the rate of tooth movement via regulation of the GSK-3β/β-catenin signaling pathway. Our results showed that continued mechanical loading increased the distance between the first and second molar in rats. In addition, the loading force increased bone formation at the tension site, and also increased phospho-Ser9-GSK-3β expression and β-catenin signaling pathway activity. Downregulation of GSK-3β activity further increased bone parameters, including bone mineral density, bone volume to tissue volume and trabecular thickness, as well as ALP- and osterix-positive cells at tension sites during tooth movement. However, ICG-001, the β-catenin selective inhibitor, reversed the positive effects of GSK-3β inhibition. In addition, pharmaceutical inhibition of GSK-3β or local treatment with β-catenin inhibitor did not influence the rate of tooth movement. Based on these results, we concluded that GSK-3β/β-catenin signaling contributes to the bone remodeling induced by orthodontic forces, and can be used as a potential therapeutic target in clinical dentistry.

## Introduction

Orthodontic tooth movement (OTM) occurs through controlled application of mechanical forces to the tooth, which leads to required changes in the periodontal ligament and alveolar bone and subsequently activates remodeling of the alveolar bone (Verna et al. 1999; Nakamura et al. 2008; Tsuge et al. 2016; Sun et al. 2017). The periodontal ligament, a connective mechanosensitive fibrous soft tissue located between the root and the alveolar bone, mediates the loading force from tooth to alveolar bone, and thus creates the necessary conditions for cells to participate in alveolar bone remodeling (Liu et al. 2013; Mabuchi et al. 2002; Cui et al. 2016). This process includes bone resorption on the pressure side and bone formation on the tension side of the alveolar bone and periodontal ligament, which together causes OTM (Verna et al. 1999; Nakamura et al. 2008; Chung et al. 2007). New bone formation on the tension side is extremely critical for the long-term stability of the moved tooth. However, the molecular mechanisms underlying the tension force-induced bone formation are poorly understood.

Recently, glycogen synthase kinase 3 (GSK-3) has been demonstrated to play an important role in multiple biological processes, including bone formation and remodeling (Geng et al. 2015; Marsell et al. 2012; Jang et al. 2011; Clevers and Nusse 2012; Clément-Lacroix et al. 2005). GSK-3, the key regulator of glycogen metabolism, is a serine/threonine kinase, which includes two kinds of isoforms, GSK-3α and GSK-3β. Although GSK-3α has 97% amino-acid sequence similarity in its kinase catalytic domains with GSK-3β, GSK-3β is more important for bone remodeling, as demonstrated by increased bone formation in GSK-3β+/− mice (Noh et al. 2009). Pharmacological inhibition of GSK-3β promotes the expression of osteogenic differentiation related factors, including osterix, runt-related transcription factor 2 (Runx2) and alkaline phosphatase (ALP) (Geng et al. 2015; Marsell et al. 2012; Krause et al. 2010), which have been demonstrated to be involved in alveolar bone formation at the tension site of the moved tooth (Chung et al. 2007; Fu et al. 2016; Kariya et al. 2015). In fact, inhibition of GSK-3β is necessary for nuclear accumulation and translocation of β-catenin (Clevers and Nusse 2012). It’s known that β-catenin is an important regulator for canonical Wnt pathway activation, which is essential for proper bone development, and downregulation of this pathway impairs bone formation (Gordon and Nusse 2006; Zhong et al. 2012). Clinical evidence of pharmacological antagonists of GSK-3β treatment showed that the canonical Wnt pathway increases bone mass through the β-catenin pathway (Vestergaard et al. 2005). All these data indicate that GSK-3β is critical for the regulation of bone formation. However, to our knowledge, there are no studies regarding the role of the GSK-3β/β-catenin signaling pathway in tension force-induced bone formation during OTM.

Therefore, in the current study, we hypothesized that orthodontic force may stimulate bone formation at the tension site of the moved tooth and increase the rate of tooth movement via regulation of the GSK-3β/β-catenin signaling pathway. We established a rat tooth movement model to test this hypothesis. This study will help us to better understand the mechanism of periodontal tissue bone remodeling during OTM, and provide us with a candidate therapeutic target for improving orthodontic treatment.

## Materials and methods

### Agents

Lithium chloride was purchased from Sigma-Aldrich (St. Louis, MD, USA). ICG-001 was provided by Tocis (Bioscience, Bristol, UK). Primary antibodies against GSK-3β, phospho-Ser9-GSK-3β, β-catenin, ALP and osterix were purchased from Abcam (Shanghai, China). Nickel-titanium (NiTi) coil springs were obtained from Tomy Incorporated (Fukushima, Japan).

### Experimental animals

All animals used in the current study were treated according to the principles and procedures approved by the Institutional Animal Ethics Committee of the First Affiliated Hospital of Soochow University. Thirty-five 8-week-old Sprague Dawley rats weighing approximately 200–250 g were obtained from the Laboratory Animal Research Center of Soochow University.

### Experimental tooth movement model and drug treatment

The orthodontic tooth movement model was established as previously described (Gu et al. 2017a, b; Liu et al. 2017a, b, c). Briefly, the rats were divided into five groups: PBS control (sham, n = 5), OTM with PBS treatment (OTM, n = 15), OTM and LiCl (LiCl group, n = 5), OTM and ICG-001 (ICG group, n = 5), and OTM, LiCl and ICG-001 (LiCl + ICG group, n = 5). The rats were anesthetized using an intraperitoneal injection of 3 mg/ml 10% chloral hydrate. A closed NiTi coil spring (50-g force) was applied between the upper incisor and the first left molar in the OTM, LiCl and ICG groups. Rats in the LiCl and LiCl + ICG groups were gavage-fed with LiCl at 200 mg/kg/day. The dosage of LiCl adopted in the current study was previously demonstrated to show bone protective effects in vivo (Clément-Lacroix et al. 2005; Gu et al. 2017a, b). Rats in the ICG group received a local injection of ICG-001 (10 μg) on the mesial-palatal aspect of the left maxillary first molar into the mucoperiosteum daily until sacrifice. In addition, the LiCl-treated rats received a 10-μL injection of PBS or ICG-001 (10 μg) at the surgery site prior to orthodontic force application and then daily until sacrifice. Rats in the sham and OTM groups received PBS daily. No adverse effects or mortality occurred during the experiment. Rats in the OTM groups were sacrificed at day 0, 7 and 14 (n = 5 at each time point) after surgery. At the end of this experiment, five rats in each group were sacrificed and the whole maxillae including the left first, second and third molars were dissected for micro-computed tomography (Micro-CT), and histological analysis.

### Micro-CT scanning

The dissected maxilla was scanned with a high-resolution Micro-CT scanner (SkyScan 1176; SkyScan, Knotich, Belgium) as previously described (Gu et al. 2017a, b; Wang et al. 2014). The maxilla was scanned at a resolution of 18 μm operating at a source voltage of 80 kV and 100 μA with an exposure time of 100 ms. After scanning, three-dimensional (3D) images were reconstructed using the manufacturer’s software. For quantitative analysis, the region of interest (ROI) was defined as the area of alveolar bone at the distal side near the mesiobuccal root, with the top surface of the cube (500 μm × 500 μm × 500 μm) located at the junction between the intermediate and apical one-thirds of the root. Parameters including bone mineral density (BMD), bone volume to tissue volume (BV/TV), trabecular separation (Tb.Sp) and trabecular thickness (Tb.Th) for the ROI were analyzed using five consecutive images.

### Histology and immunohistochemistry

After Micro-CT scanning, the maxillae were dissected and trimmed into smaller blocks containing the first molars. All blocks were decalcified in 10% (w/v) ethylene diamine tetraacetic acid for 3 weeks, and then embedded in paraffin. The blocks were cross-sectioned to 5 μm, mounted on protein-coated glass slides, dewaxed, and stained with hematoxylin and eosin (HE). Using a magnification of ×20, digital photographs centered on the midline suture of the sections were taken.

For immunohistological staining, the deparaffinized sections were incubated with primary rabbit-anti-mouse antibodies, including GSK-3β antibody (1:200, ab32391), phosphor-Ser9-GSK-3β antibody (1:250, ab75814), β-catenin antibody (1:500, ab32572), ALP antibody (1:500, ab108337) and osterix antibody (1:100, ab22552) at 4 °C. After washing, the sections were incubated with biotin-conjugated secondary antibodies and then avidin–biotin enzyme reagents for 30 min. Staining was visualized by using 3.3′-diaminobezidine tetrahydrochloride as the substrate chromogen and hematoxylin as a counterstain. Positive staining was determined microscopically and counted by two independent observers.

### Statistical analysis

Data were expressed as mean ± standard deviation. One-way analysis of variance with Tukey post-hoc pairwise comparisons were used for evaluating statistical significance. All *P* values < 0.05 were considered statistically significant.

## Results

### Bone formation induced by tension force during tooth movement

**Fig. 1:**
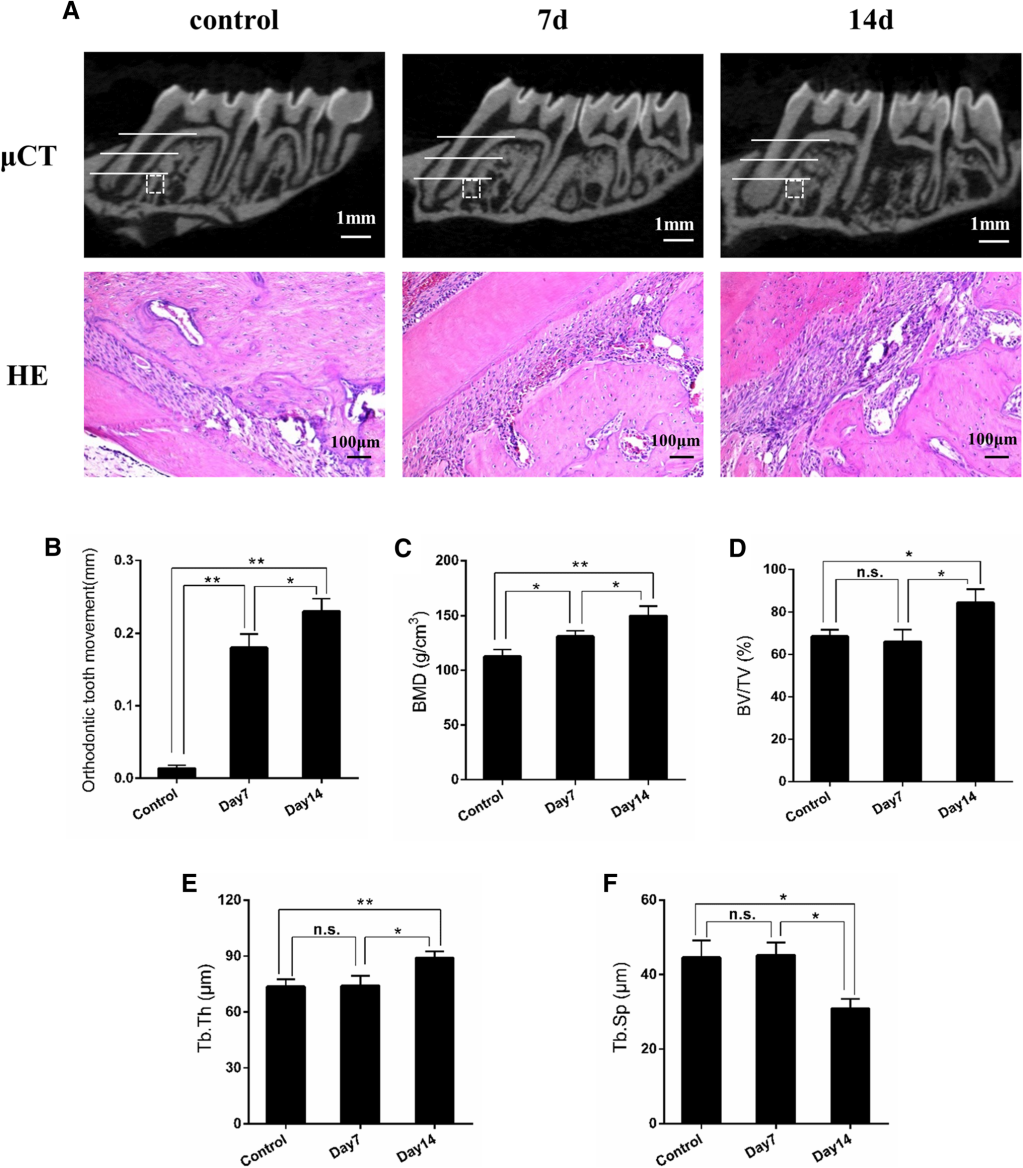
Orthodontic force loading promotes tooth movement and induces bone formation at tension sites. **a** Shown are the Micro-CT (scale bar = 1 mm) and HE staining (scale bar = 100 μm) images of the moved tooth. The distance of orthodontic tooth movement (**b**), BMD (**c**), BV/TV (**d**), Tb.Th and Tb.Sp were calculated. n = 5 per group. *P < 0.05, **P < 0.01. *n.s* No significance

As shown in Fig. 1a, the first molar moved mesially after orthodontic force loading. Furthermore, Micro-CT analysis of the alveolar bone at the tension site also showed that the examined bone parameters, including BMD, BV/TV, and Tb.Th, were significantly increased, while Tb.Sp decreased markedly after orthodontic force stimulation (Fig. 1c–f). Quantification analysis showed that the distance between the first and second molar was significantly greater in mechanical loading-induced rats than in control groups at 14 days after surgery (0 vs. 0.24 mm, *P* < 0.01; Fig. 1b). A wider periodontal space was observed in the experimental rats than in the control group, which is consistent with the Micro-CT results.

**Fig. 2:**
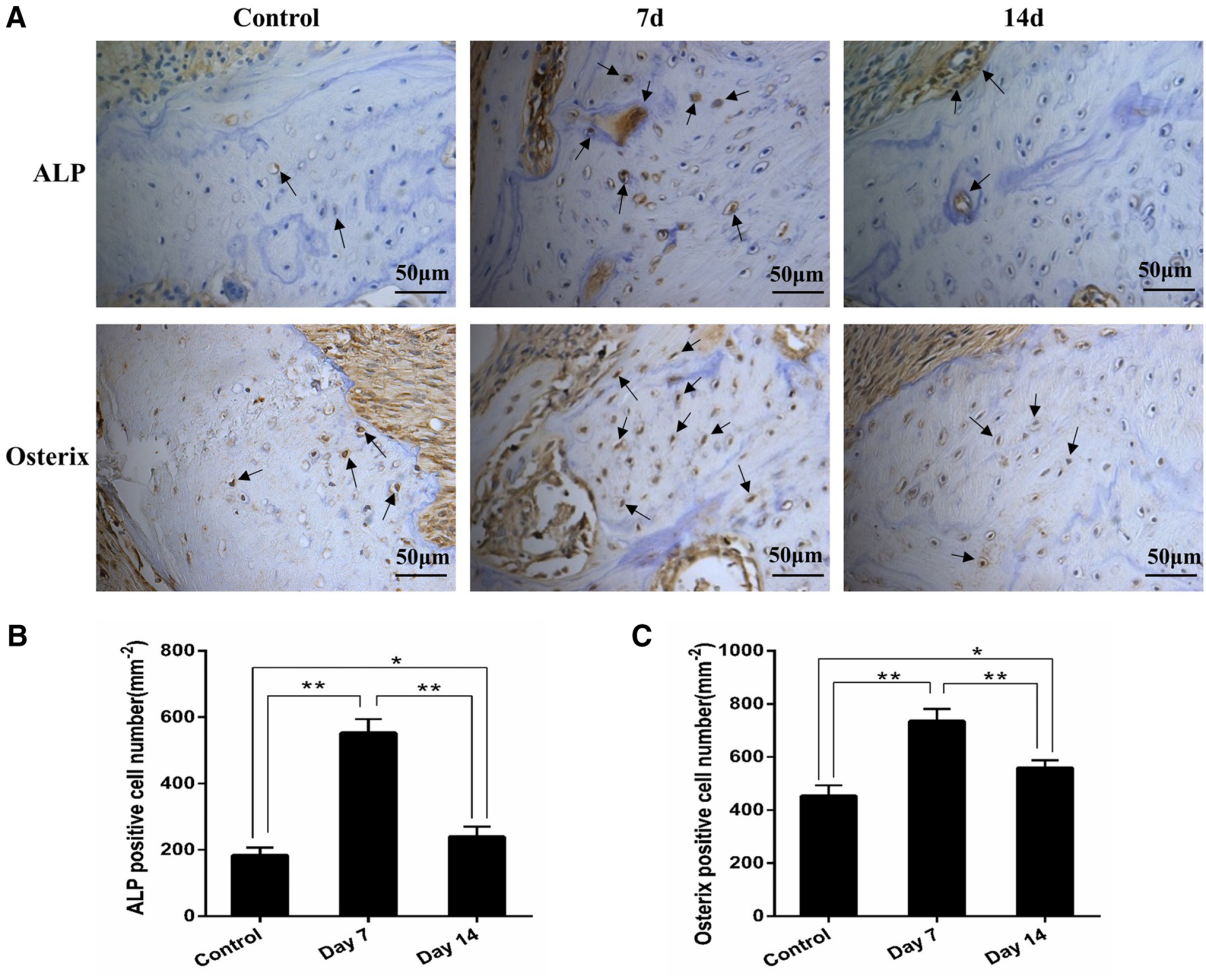
ALP and osterix expression at the tension site during tooth movement. **a** Immunohistochemical detection of ALP and osterix at the tension site of the moved tooth (scale bar = 50 μm). **b, c** Semiquantitative analysis by two independent pathologists was performed. *P < 0.05, **P < 0.01

Immunohistochemical analysis further supported the concept that mechanic loading induced bone formation during tooth movement. As shown in Fig. 2, more ALP and osterix positive cells, which are commonly used for new bone formation, were observed at the tension site in the mechanic loading-induced rats compared with the control group. These results demonstrated that mechanical force induced bone formation at the tension site and promoted the rate of tooth movement during OTM.

### Orthodontic tooth movement and the GSK-3β/β-catenin signaling pathway

**Fig. 3:**
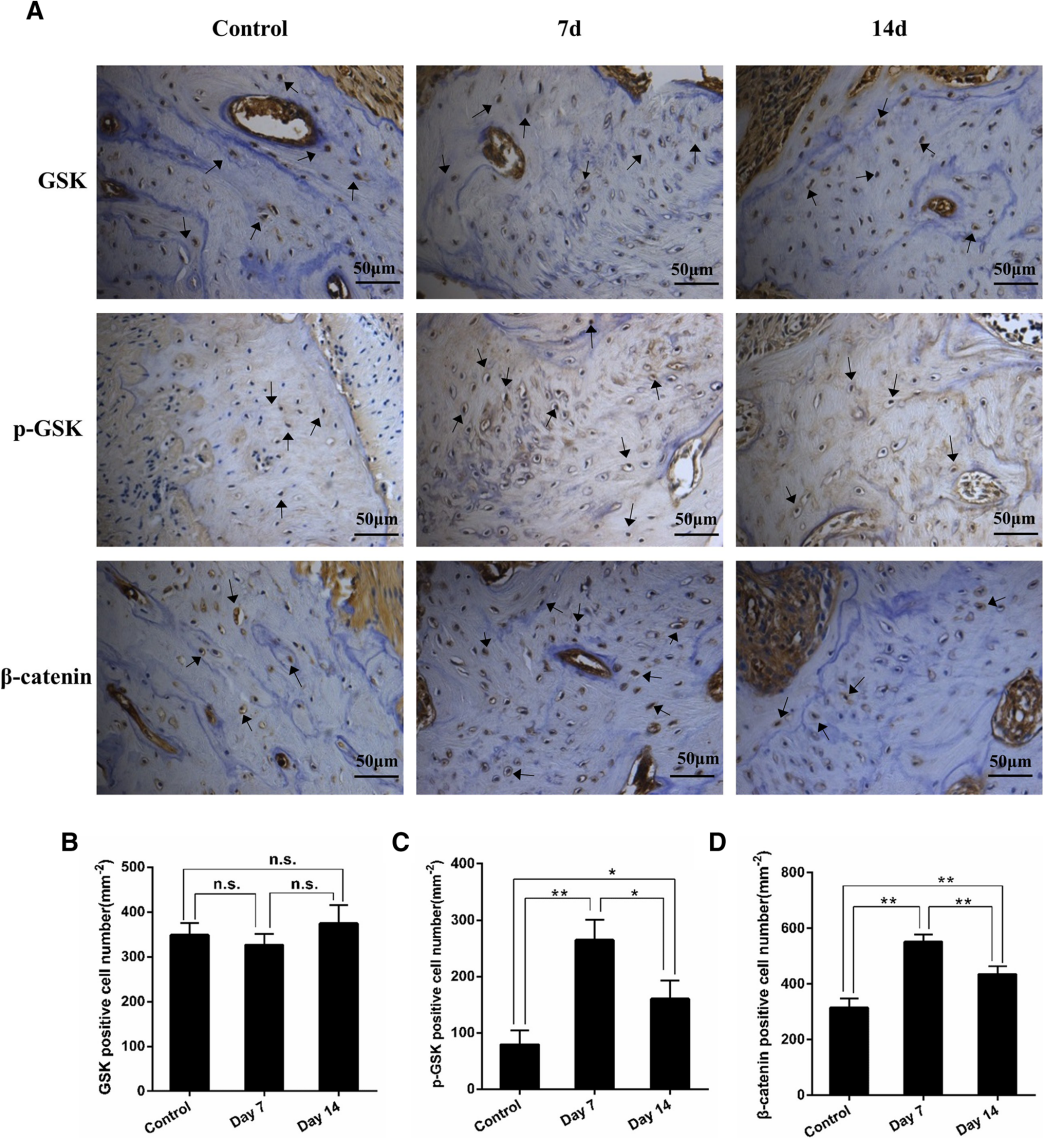
Orthodontic force loading suppressed GSK-3β activity and promoted β-catenin expression at the tension site during tooth movement. **a** Representative immunohistochemical images of GSK-3β, phosphor-Ser9-GSK-3β and β-catenin (brown, indicated by the arrows, scale bar = 50 μm). **b**–**d** Semiquantitative analysis by two independent pathologists was performed and showed that orthodontic force significantly increased the expression of phosphor-Ser9-GSK-3β and β-catenin, but not GSK-3β at the tension site of the moved tooth. *P < 0.05, **P < 0.01. *n.s* no significance

As shown in Fig. 3a, intense phospho-Ser9-GSK-3β staining was observed in force loading-induced rats compared with the low expression found in the control group. However, mechanic loading had little effect on the level of GSK-3β staining in rats (Fig. 3b). The semi-quantitative analysis indicated that loading force significantly increased the expression of phospho-Ser9-GSK-3β at day 7; the level then declined at day 14, but was still higher than that in untreated rats (Fig. 3c). Considering the important role of GSK-3β on β-catenin expression, we next determined the expression of β-catenin in force loading-induced rats. Immunohistological staining demonstrated that mechanical loading significantly increased the expression of β-catenin (Fig. 3d), which indicated that tension force activates the GSK-3β/β-catenin signaling pathway at the tension site of the moved tooth.

### The GSK-3β/β-catenin signaling pathway mediates tension force-induced bone formation during OTM

**Fig. 4:**
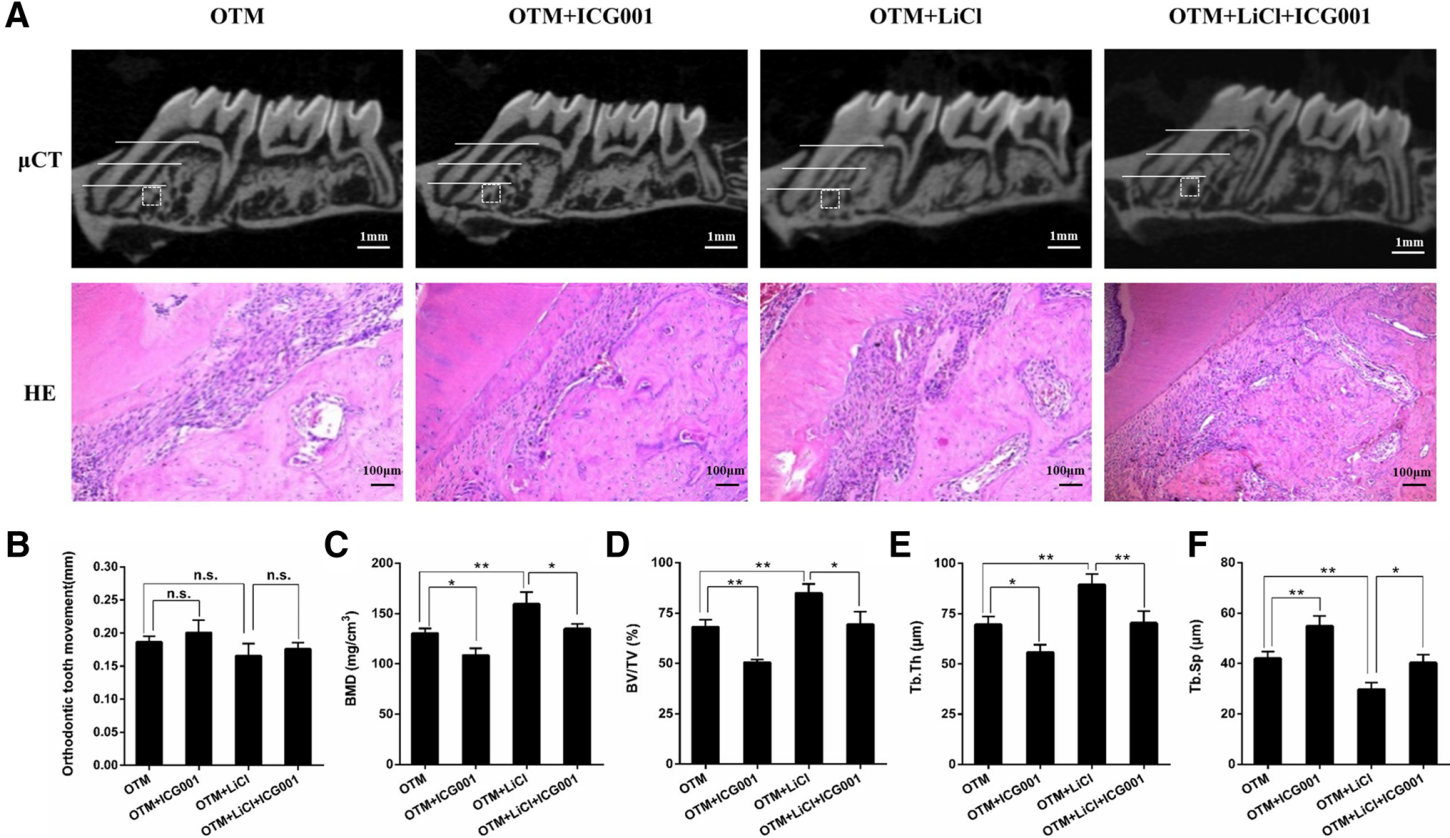
Pharmaceutical inhibition of GSK-3β promoted tension force-induced bone formation. **a** Micro-CT (scale bar = 1 mm) and HE (scar bar = 100 μm) images in maxillae of rats. The distance of orthodontic tooth movement (**b**), BMD (**c**), BV/TV (**d**), Tb.Th (**e**) and Tb.Sp (**f**) were calculated. n = 5 per group. *P < 0.05, **P < 0.01. *n.s* No significance

**Fig. 5:**
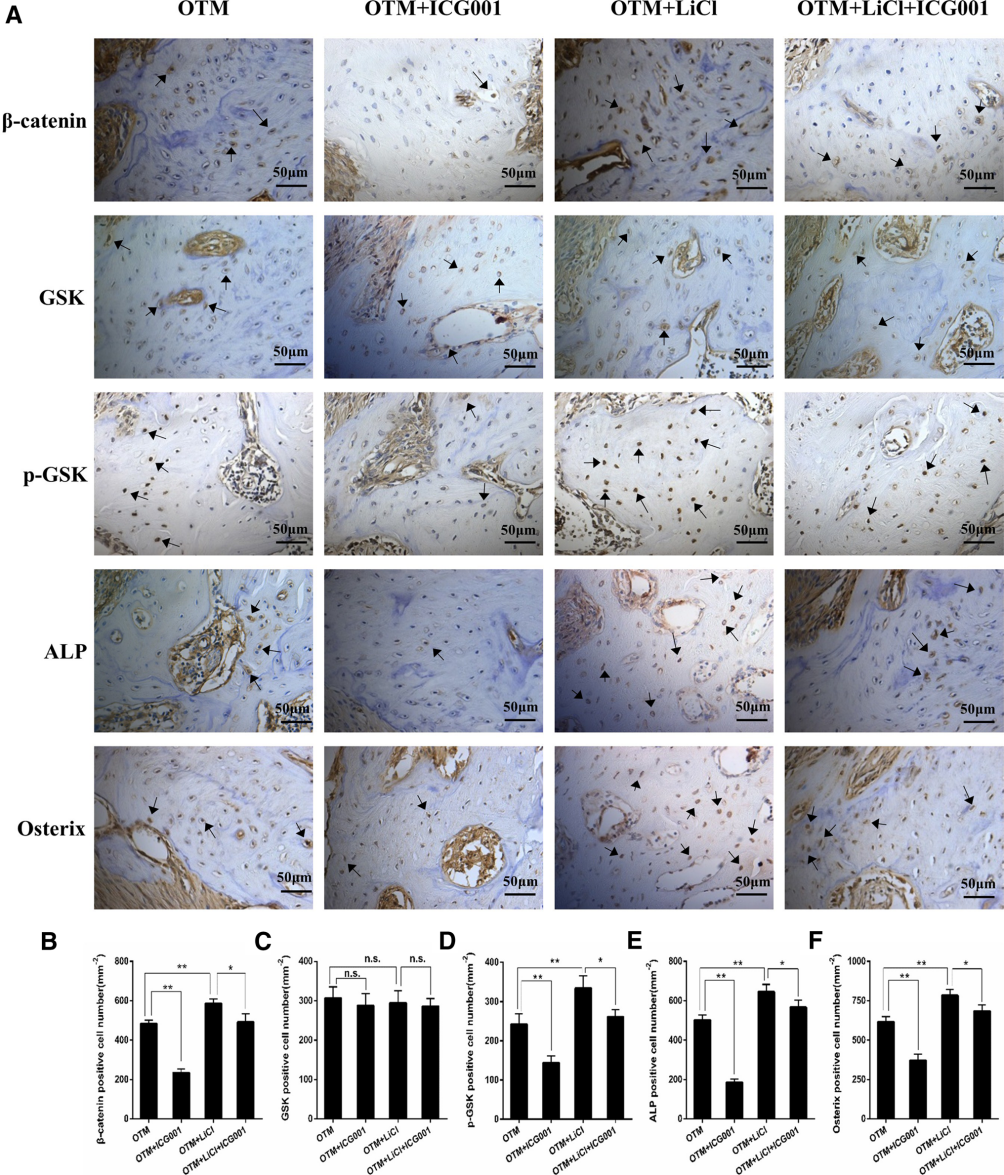
Immunohistochemical staining for GSK-3β, phosphor-Ser9-GSK-3β, β-catenin, ALP and osterix at the tension site of the moved tooth (**a** brown, indicated by arrows, scale bar = 50 μm) and semiquantitative analysis by two independent pathologists was performed (**b**–**f**). *P < 0.05, **P < 0.01. *n.s* No significance. (Color figure online)

To investigate whether loading force induced bone formation through regulation of the GSK-3β/β-catenin signaling pathway in vivo, firstly, the loading force-induced rats were gavage fed with GSK-3β selective inhibitor for 7 days. As shown in Fig. 4, downregulation of GSK-3β significantly increased BMD within a ROI of alveolar bone at the tension site (132.3 vs. 160.8 mg/cm^3^; *P* < 0.01). Micro-CT also revealed a marked increase in BV/TV and Tb.Th, and a marked decrease in Tb.Sp in rats stimulated with GSK-3β selective inhibitor, consistent with the increased new bone deposition shown in HE staining. To determine whether the increased BMD was caused by increased osteoblast function, immunohistological staining was used to detect ALP and osterix expression. As expected, daily gavage feeding with the GSK-3β inhibitor evidently increased ALP- and osterix-positive cells in the ROI of alveolar bone at the tension site (Fig. 5).

Because previous studies have demonstrated that GSK-3β is an essential regulator for the β-catenin signaling pathway (Marsell et al. 2012; Clevers and Nusse 2012), we investigated whether GSK-3β inhibitor promotes loading force-induced bone formation via the β-catenin signaling pathway. As expected, lithium chloride significantly increased the expression of phospho-Ser9-GSK-3β compared with that in untreated rats. Additionally, many β-catenin positive cells were observed in the GSK-3β inhibitor treatment groups (Fig. 5a, b, d). These data indicate that lithium chloride inhibits GSK-3β and subsequently activates the β-catenin signaling pathway at tension sites during OTM.

The number of β-catenin positive cells decreased markedly when ICG-001, a β-catenin inhibitor, was administered to rats that have undergone OTM and lithium chloride-treated, further indicating that lithium chloride activates the β-catenin signaling pathway at tension sites. Additionally, local treatment with ICG-001 attenuated the effects of lithium chloride on bone mass and on ALP and osterix expression (Fig. 5a, b, e, f), which indicates that GSK-3β inhibitor promotes new bone formation via the β-catenin signaling pathway at tension sites during tooth movement.

### The GSK-3β/β-catenin signaling pathway and the rate of tooth movement

We next investigated whether modulating the GSK-3β/β-catenin signaling pathway affects the distance between the first and second molar in mechanical loading-induced rats. Our results showed that pharmaceutical inhibition of GSK-3β did not restrict the rate of tooth movement during OTM (0.1889 vs. 0.1673 mm, *P* > 0.05). However, local treatment with ICG-001 also showed less impact on orthodontic force-induced tooth movement (Fig. 4a, b). These data demonstrate that modulating the activity of the GSK-3β/β-catenin signaling pathway did not influence the rate of tooth movement.

## Discussion

OTM is dependent on the efficient remodeling of periodontal tissue, especially alveolar bone, induced by proper mechanical loading (Verna et al. 1999; Nakamura et al. 2008; Tsuge et al. 2016). To understand the biological relationship between orthodontic force and OTM, several authors previously explored the role of osteoclasts stimulated with orthodontic force (Pazzini et al. 2017; Liu et al. 2017a, b, c; Wang et al. 2017). However, mechanical stimulation not only directly affects bone homeostasis by stimulating osteoclastogenesis, but also has positive effects on osteoblastogenesis. Recent studies demonstrated that marked upregulation of bone formation from increased osteoblast activity was a common feature of OTM at tension sites (Fu et al. 2016; Kariya et al. 2015; Liu et al. 2017a, b, c; Dai et al. 2017). However, the mechanism of orthodontic force-induced bone formation at the tension site of a moved tooth is unclear. Our results showed that proper mechanical loading significantly increased levels of ALP and osterix-positive cells, which is consistent with previous studies (Fu et al. 2016; Dai et al. 2017). Additionally, mechanical loading obviously increased bone volume at tension sites in the rat tooth movement model. These effects were related to suppression of GSK-3β and further activation of the β-catenin signaling pathway. These results support the concept that the GSK-3β/β-catenin pathway plays an important role in bone formation at tension sites during OTM.

Osteoblasts, the main cells in bone tissue and bone formation, are critical for alveolar bone morphology and the stability of the moved tooth (Aksu et al. 2010). Continuous exposure to mechanical force during orthodontic treatment promotes the function of mature osteoblasts, as well as increasing bone formation and differentiation of osteoblast precursors (Fu et al. 2016; Dai et al. 2017; Qin and Hua 2016), which is in line with the results of the current study. It’s believed that bone formation is significantly increased at tension sites during orthodontic treatment, which is partially responsible for the effect of OTM. The GSK-3β/β-catenin signaling pathway regulates osteoblast differentiation and bone formation in vivo (Geng et al. 2015; Marsell et al. 2012), and there is increasing data to suggest a role for this pathway in mechanical loading circumstances (Premaraj et al. 2011; Chen et al. 2013). In support of this, we demonstrated a clear increase in pSer9-GSK-3β and β-catenin expression at the tension site of the moved tooth, suggesting that these proteins increased osteoblastogenesis by modulating the GSK-3β/β-catenin signaling pathway in osteoblasts.

GSK-3β is known to be involved in the regulation of bone homeostasis. We provided evidence that GSK-3β mediated the deposition of new bone at the tension site of the moved tooth. Previous studies demonstrated that heterozygous GSK-3β-deficient mice display a higher bone formation rate, higher bone mass and more osteoblasts per bone surface (Noh et al. 2009). Pharmacological inhibition of GSK-3β reduced ovariectomy-induced bone loss and enhanced bone mineral density in wild type animals (Krause et al. 2010). In the current study, we demonstrated that mechanical loading significantly decreased GSK-3β activity, which is negatively related to orthodontic force-induced bone formation at tension sites in OTM. More interestingly, morphometric analysis revealed even higher BMD, BV/TV and Tb.Th in GSK-3β inhibitor-treated animals compared with untreated rats. These results strongly suggest that GSK-3β plays an important role in mechanical loading-induced bone formation.

β-catenin is a critical regulator for canonical Wnt pathway activation, which is essential for bone homeostasis, and downregulation of this pathway suppresses new bone formation (Gordon and Nusse 2006; Zhong et al. 2012). Previous reports showed that β-catenin activity is tightly controlled by N-terminal phosphorylation by GSK-3β. When GSK-3β is phosphorylated at Ser9, its ability to phosphorylate β-catenin is blocked (Clevers and Nusse 2012). Our results showed that mechanical loading markedly increased phospho-Ser9-GSK-3β and β-catenin expression. Meanwhile, GSK-3β inhibitor treatment significantly increased the expression of β-catenin, and subsequently increased bone formation at the tension site of the moved tooth. However, administration of β-catenin inhibitor significantly reversed these effects. These results indicate that mechanical loading induced bone formation at tension sites by the GSK-3β/β-catenin signaling pathway.

Proper mechanical loading reduced GSK-3β activity and increased β-catenin expression, thus inducing bone formation at tension sites during OTM. Our results showed that consistent orthodontic force significantly increased the distance between the left first and second molars, which is consistent with previous reports (Gu et al. 2017a, b; Liu et al. 2017a, b, c). Modulating the GSK-3β/β-catenin signaling pathway influences mechanical loading-induced bone formation at tension sites, but did not affect the rate of tooth movement during OTM. Previous studies demonstrated that the rate of tooth movement depends on the remodeling activity of alveolar bone, which is characterized by compression force-induced bone resorption and tension force-induced bone formation (Verna et al. 1999; Nakamura et al. 2008; Chung et al. 2007). In the current study, we demonstrated the relationship between GSK-3β/β-catenin signaling and tension force-induced bone formation. However, the relationship between GSK-3β/β-catenin signaling and compression force-induced bone resorption remains unclear, and this is currently the focus of ongoing studies in our laboratory.

In summary, this study demonstrated that the activity of GSK-3β/β-catenin signaling was associated with orthodontic force-induced bone formation at tension sites during OTM. Pharmacological inhibition of GSK-3β may accelerate tension force-induced bone formation without influencing the rate of tooth movement. Collectively, our findings indicated that GSK-3β/β-catenin signaling contributes to orthodontic force-induced bone remodeling, and can be used as a potential therapeutic target in clinical dentistry.

## References

[CR1] Aksu M, Saglam-Aydinatay B, Akcan CA, El H, Taner T, Kocadereli I, Tuncbilek G, Mavili ME (2010). Skeletal and dental stability after maxillary distraction with a rigid external device in adult cleft lip and palate patients. J Oral Maxillofac Surg.

[CR2] Chen X, Hu C, Wang G, Li L, Kong X, Ding Y, Jin Y (2013). Nuclear factor-κB modulates osteogenesis of periodontal ligament stem cells through competition with β-catenin signaling in inflammatory microenvironments. Cell Death Dis.

[CR3] Chung CJ, Baik HS, Soma K (2007). Bone formation and tooth movement are synergistically enhanced by administration of EP4 agonist. Am J Orthod Dentofacial Orthop.

[CR4] Clément-Lacroix P, Ai M, Morvan F, Roman-Roman S, Vayssière B, Belleville C, Estrera K, Warman ML, Baron R, Rawadi G (2005). Lrp5-independent activation of Wnt signaling by lithium chloride increases bone formation and bone mass in mice. Proc Natl Acad Sci USA.

[CR5] Clevers H, Nusse R (2012). Wnt/β-catenin signaling and disease. Cell.

[CR6] Cui J, Li J, Wang W, Han X, Du J, Sun J, Feng W, Liu B, Liu H, Amizuka N, Li M (2016). The effect of calcitriol on high mobility group box 1 expression in periodontal ligament cells during orthodontic tooth movement in rats. J Mol Histol.

[CR7] Dai Q, Zhou S, Zhang P, Ma X, Ha N, Yang X, Yu Z, Fang B, Jiang L (2017). Force-induced increased osteogenesis enables accelerated orthodontic tooth movement in ovariectomized rats. Sci Rep.

[CR8] Fu HD, Wang BK, Wan ZQ, Lin H, Chang ML, Han GL (2016). Wnt5a mediated canonical Wnt signaling pathway activation in orthodontic tooth movement: possible role in the tension force-induced bone formation. J Mol Histol.

[CR9] Geng D, Wu J, Shao H, Zhu S, Wang Y, Zhang W, Ping Z, Hu X, Zhu X, Xu Y, Yang H (2015). Pharmaceutical inhibition of glycogen synthetase kinase 3 beta suppresses wear debris-induced osteolysis. Biomaterials.

[CR10] Gordon MD, Nusse R (2006). Wnt signaling: multiple pathways, multiple receptors, and multiple transcription factors. J Biol Chem.

[CR11] Gu Q, Guo S, Wang D, Zhou T, Wang L, Wang Z, Ma J (2017). Effect of corticision on orthodontic tooth movement in a rat model as assessed by RNA sequencing. J Mol Histol.

[CR12] Gu Y, Wang Z, Shi J, Wang L, Hou Z, Guo X, Tao Y, Wu X, Zhou W, Liu Y, Zhang W, Xu Y, Yang H, Xue F, Geng D (2017). Titanium particle-induced osteogenic inhibition and bone destruction are mediated by the GSK-3β/β-catenin signal pathway. Cell Death Dis.

[CR13] Jang HD, Shin JH, Park DR, Hong JH, Yoon K, Ko R, Ko CY, Kim HS, Jeong D, Kim N, Lee SY (2011). Inactivation of glycogen synthase kinase-3β is required for osteoclast differentiation. J Biol Chem.

[CR14] Kariya T, Tanabe N, Shionome C, Manaka S, Kawato T, Zhao N, Maeno M, Suzuki N, Shimizu N (2015). Tension force-induced ATP promotes osteogenesis through P2 × 7 receptor in osteoblasts. J Cell Biochem.

[CR15] Krause U, Harris S, Green A, Ylostalo J, Zeitouni S, Lee N, Gregory CA (2010). Pharmaceutical modulation of canonical Wnt signaling in multipotent stromal cells for improved osteoinductive therapy. Proc Natl Acad Sci USA.

[CR16] Liu O, Xu J, Ding G, Liu D, Fan Z, Zhang C, Chen W, Ding Y, Tang Z, Wang S (2013). Periodontal ligament stem cells regulate B lymphocyte function via programmed cell death protein 1. Stem Cells.

[CR17] Liu Y, Zhang T, Zhang C, Jin SS, Yang RL, Wang XD, Jiang N, Gan YH, Kou XX, Zhou YH (2017). Aspirin blocks orthodontic relapse via inhibition of CD4 + T lymphocytes. J Dent Res.

[CR18] Liu F, Wen F, He D, Liu D, Yang R, Wang X, Yan Y, Liu Y, Kou X, Zhou Y (2017). Force-Induced H2S by PDLSCs Modifies Osteoclastic Activity during Tooth Movement. J Dent Res.

[CR19] Liu L, Liu M, Li R, Liu H, Du L, Chen H, Zhang Y, Zhang S, Liu D (2017). MicroRNA-503-5p inhibits stretch-induced osteogenic differentiation and bone formation. Cell Biol Int.

[CR20] Mabuchi R, Matsuzaka K, Shimono M (2002). Cell proliferation and cell death in periodontal ligaments during orthodontic tooth movement. J Periodontal Res.

[CR21] Marsell R, Sisask G, Nilsson Y, Sundgren-Andersson AK, Andersson U, Larsson S, Nilsson O, Ljunggren O, Jonsson KB (2012). GSK-3 inhibition by an orally active small molecule increases bone mass in rats. Bone.

[CR22] Nakamura Y, Noda K, Shimoda S, Oikawa T, Arai C, Nomura Y, Kawasaki K (2008). Time-lapse observation of rat periodontal ligament during function and tooth movement, using microcomputed tomography. Eur J Orthod.

[CR23] Noh T, Gabet Y, Cogan J, Shi Y, Tank A, Sasaki T, Criswell B, Dixon A, Lee C, Tam J, Kohler T, Segev E, Kockeritz L, Woodgett J, Müller R, Chai Y, Smith E, Bab I, Frenkel B (2009). Lef1 haploinsufficient mice display a low turnover and low bone mass phenotype in a gender- and age-specific manner. PLoS ONE.

[CR24] Pazzini CA, Pereira LJ, da Silva TA, Montalvany-Antonucci CC, Macari S, Marques LS, de Paiva SM (2017). Probiotic consumption decreases the number of osteoclasts during orthodontic movement in mice. Arch Oral Biol.

[CR25] Premaraj S, Souza I, Premaraj T (2011). Mechanical loading activates β-catenin signaling in periodontal ligament cells. Angle Orthod.

[CR26] Qin J, Hua Y (2016). Effects of hydrogen sulfide on the expression of alkaline phosphatase, osteocalcin and collagen type I in human periodontal ligament cells induced by tension force stimulation. Mol Med Rep.

[CR27] Sun J, Du J, Feng W, Lu B, Liu H, Guo J, Amizuka N, Li M (2017). Histological evidence that metformin reverses the adverse effects of diabetes on orthodontic tooth movement in rats. J Mol Histol.

[CR28] Tsuge A, Noda K, Nakamura Y (2016). Early tissue reaction in the tension zone of PDL during orthodontic tooth movement. Arch Oral Biol.

[CR29] Verna C, Zaffe D, Siciliani G (1999). Histomorphometric study of bone reactions during orthodontic tooth movement in rats. Bone.

[CR30] Vestergaard P, Rejnmark L, Mosekilde L (2005). Reduced relative risk of fractures among users of lithium. Calcif Tissue Int.

[CR33] Wang Y, Gao S, Jiang H, Lin P, Bao X, Zhang Z, Hu M (2014). Lithium chloride attenuates root resorption during orthodontic tooth movement in rats. Exp Ther Med.

[CR31] Wang C, Gu W, Sun B, Zhang Y, Ji Y, Xu X, Wen Y (2017). CTHRC1 promotes osteogenic differentiation of periodontal ligament stem cells by regulating TAZ. J Mol Histol.

[CR32] Zhong Z, Zylstra-Diegel CR, Schumacher CA, Baker JJ, Carpenter AC, Rao S, Yao W, Guan M, Helms JA, Lane NE, Lang RA, Williams BO (2012). Wntless functions in mature osteoblasts to regulate bone mass. Proc Natl Acad Sci USA.

